# Does substrate matter in the deep sea? A comparison of bone, wood, and carbonate rock colonizers

**DOI:** 10.1371/journal.pone.0271635

**Published:** 2022-07-20

**Authors:** Olívia S. Pereira, Jennifer Gonzalez, Guillermo Mendoza, Jennifer Le, Madison McNeill, Jorge Ontiveros, Raymond W. Lee, Greg W. Rouse, Jorge Cortés, Lisa A. Levin

**Affiliations:** 1 Scripps Institution of Oceanography, University of California, San Diego, San Diego, California, United States of America; 2 College of Health and Sciences, East Central University, Ada, Oklahoma, United States of America; 3 Instituto Tecnológico de Tijuana, Tijuana, Mexico; 4 School of Biological Sciences, Washington State University, Pullman, Washington, United States of America; 5 Centro de Investigación en Ciencias del Mar y Limnología, Universidad de Costa Rica, San Pedro, San José, Costa Rica; University of California, UNITED STATES

## Abstract

Continental margins host methane seeps, animal falls and wood falls, with chemosynthetic communities that may share or exchange species. The goal of this study was to examine the existence and nature of linkages among chemosynthesis-based ecosystems by deploying organic fall mimics (bone and wood) alongside defaunated carbonate rocks within high and lesser levels of seepage activity for 7.4 years. We compared community composition, density, and trophic structure of invertebrates on these hard substrates at active methane seepage and transition (less seepage) sites at Mound 12 at ~1,000 m depth, a methane seep off the Pacific coast of Costa Rica. At transition sites, the community composition on wood and bone was characteristic of natural wood- and whale-fall community composition, which rely on decay of the organic substrates. However, at active sites, seepage activity modified the relationship between fauna and substrate, seepage activity had a stronger effect in defining and homogenizing these communities and they depend less on organic decay. In contrast to community structure, macrofaunal trophic niche overlap between substrates, based on standard ellipse areas, was greater at transition sites than at active sites, except between rock and wood. Our observations suggest that whale- and wood-fall substrates can function as stepping stones for seep fauna even at later successional stages, providing hard substrate for attachment and chemosynthetic food.

## Introduction

Chemosynthesis-based ecosystems (CBEs) are highly productive systems that support diverse communities reliant on chemosynthesis rather than photosynthesis. They include hydrothermal vents, methane seeps, and organic falls (whale and wood falls), and are patchily distributed around the world [1]. Methane seeps are commonly found on continental margins, as are whale falls, which are also found on continental margins along whale migration routes, and wood falls, near mouths of rivers that carry fallen trees to the ocean [2, 3]. The interconnectivity of these different systems, for example through genetic exchange of individuals attracted to chemosynthetic activity, has long been of interest (e.g., [4]).

Methane seeps commonly feature authigenic carbonate rocks [5], a by-product of the anaerobic oxidation of methane carried out by a consortium of methanotrophic archaea and sulfate-reducing bacteria [6, 7]. These rocks provide substrate for settlement, attachment, refuge, reproduction, and even food supply to a diverse community of invertebrates [8]. Thus, they attract not only seep communities (composed mainly of species that are endemic to seeps, i.e., seep species, see S1 Table for definitions), but also background communities (composed mainly of species that are more commonly associated with non-seep, background areas, i.e., background species, see S1 Table). Background species include mobile predators and scavengers that feed on the chemosynthetic production at the active area of the seep and carry that chemosynthetically fixed organic matter to background communities promoting trophic interaction between seep and background communities [9]. When seepage activity ceases, these rocks persist and provide hard substrate for background species that colonize them, sustaining such communities for decades [10]. Moreover, seepage activity is dynamic, and declines in fluid flux over time and space (see [11–13]) can create a transition zone surrounding the active area, where bacterial biomass and seep species diminish [14] but the rocks formed during high seepage activity levels persist, providing hard substrate for background species [10]. Although transition zones visually appear inactive, chemosynthetic microbial activity may also still persist within the carbonates [12, 15, 16], and some seep species can still be found on these rocks (see [11, 13]). Thus, transition zones can be highly diverse, with both seep and background species coexisting, enhancing habitat and trophic complexity [13, 17]. See S1 Table for a summary of terminology and definitions.

Transition zones can also provide potential connectivity among CBEs. Estimates based on biophysical modeling and population genetics have shown that deep-sea dispersal probably exceeds the average sphere of influence of the average chemosynthetic site reaching background areas [9]. Although some key abiotic features such as temperature and sulfide concentrations might differ among these, they can share both opportunistic and specialist species, suggesting the existence of a faunal network across ocean basins connected by larval dispersal stages [2]. Organic falls such as whale- and wood-falls may function as stepping stones for vent and seep species, as they create ephemeral ecosystems that attract chemosynthetic species [2, 18–20]. Likewise, carbonate rocks at transition zones may then promote a link among CBEs extending the limits of the seep for the reasons described above.

Colonization experiments with carbonate rocks and organic parcels have shown that chemosynthesis-based communities are able to rapidly colonize hard substrates on continental margins. Carbonate rocks deployed at methane seeps for 10.5 months [21] and 7.4 years [13] are colonized by seep species even at transition sites (i.e., a site within the transition zone), these species coexist with the background fauna. At hydrothermal vents, increasing hydrothermal activity supports higher functional diversity of colonizers on rocks deployed for 2 years close to active sites, although there was faunal overlap between rocks at active site and inactive sites and export of chemosynthetic production to colonizers at inactive sites [22]. A colonization experiment in the Northeast Pacific showed that co-located wood- and whale-fall substrates support high-species assemblages after only 15 months, promoting biodiversity in the deep sea [23]. Wood and bone deployed on the Southwest Atlantic Ocean for 16 months recovered an alvinocaridid shrimp species [24] and a new species of the abyssochrysoid snail *Cordesia* [25], that were both species previously known only from seeps and represent families that have been reported only from CBEs (see [25, 26]). Although the discovery of seeps in the Southwest Atlantic Ocean remains incomplete [27], pockmarks hosting seep communities have been reported [28], as well as a chemosynthetic community off the coast of southern Brazil associated with high-levels of methane and gas hydrates [29]. These findings suggest the existence of other deep-sea CBEs in the region that could function as larval sources to these ephemeral organic-fall systems and vice versa [24].

In addition to the connectivity among CBEs based on taxonomic composition, transition zones can also provide a pathway for transfer of chemosynthetic production through the horizontal advection of particulate organic matter [8]. The contribution of chemosynthetic organic matter to animal diets can be assessed through stable isotope (usually carbon and nitrogen) analyses [30, 31]. The carbon isotope composition is an excellent indicator of carbon source at the base of the food web [32, 33], where seep-associated chemosynthetic sources typically have lower δ^13^C values than photosynthetic sources [34]. The value of the nitrogen isotope at the base of the food chain at seeps is heavily influenced by local N_2_ fixation, and typically increases with trophic level; the lower the δ^15^N, the higher the contribution of locally fixed N_2_ [35] and the lower the trophic level. A species’ stable isotope composition indicates its trophic niche, and community-wide metrics describe the trophic structure of the entire species assemblage [36].

The goal of this study was to compare the communities of macrofauna on organic falls (bone and wood) relative to seep communities on carbonate rocks to assess the existence and nature of system linkages among CBEs. This was done by deploying organic fall mimics (wood and bone) alongside defaunated carbonate rocks at active and transition sites (see Methods section for defaunation procedure). We compared community composition, density, and trophic structure of invertebrates on wood, bone and carbonate deployed for 7 years at active and transition sites at Mound 12 at ~1,000 m depth, a methane seep off the Pacific coast of Costa Rica. We hypothesized the trophic contribution (i.e., nutrition) to macrofauna from organic-rich bones and wood should dominate at transition sites with lower seepage, and the macrofaunal community should differ among bones, woods, and carbonate substrates, based on previous studies that showed that geochemical processes and species composition at species level differ among CBEs (see [2]), with organic-fall specialists colonizing the organic substrates. At active sites, seepage activity should be a strong determinant of the macrofaunal community composition and its trophic structure across different types of hard substrate [see 13]. Seepage and the abundant food it creates should override the relative importance of the substrate itself and its nutritional influence, with macrofaunal communities on carbonate rock (methane seeps), bone (animal falls) and wood (wood falls) being more similar under active seepage, and less similar at transition sites where substrate would matter. We compare our findings to other shorter-term substrate experiments deployed previously at this seep site, at other seeps, vents, and in proximity to CBEs, and discuss the linkages between organic falls and methane seeps off Costa Rica.

## Methods

### Study area

The Pacific margin of Costa Rica is an offshore convergent margin, where fluid venting has long been documented in the area with evidence for more than 100 seeps [37, 38]. These seeps are associated with landslide scars, seamount-subduction related fractures, faults, and many mounds. Over 60 mounds 50–100 m high and up to 1 km wide at the base were identified in the region [38]. Among these, Mound 12 (8°55.8’N, 84°18.7’W) is located at around 1,000 m water depth [39], below the oxygen minimum zone [11]. The benthic fauna at active seepage sites is characterized by carbonate rocks hosting the yeti crabs *Kiwa puravida* and bathymodiolin mussel beds [11, 38, 40], as well as smaller animals such as neolepetopsid limpets and provannid snails [13]. Transition sites are located at the intersection of seep and background environments (described above) and are identified by the presence of scattered seep-specialist megafauna (e.g., *Bathymodiolus* mussels and yeti crabs), shell remains of seep-associated bivalves (e.g., *Bathymodiolus* mussels and vesicomyid clams), and heterogeneous soft and hard bottom with partially buried but visible carbonate rocks [17] hosting seep species (e.g., various gastropod molluscs and annelid worms) and background species (e.g., amphipods, ophiuroids), which are more commonly found at seep and background sites, respectively [13].

### Experimental design

**Fig 1:**
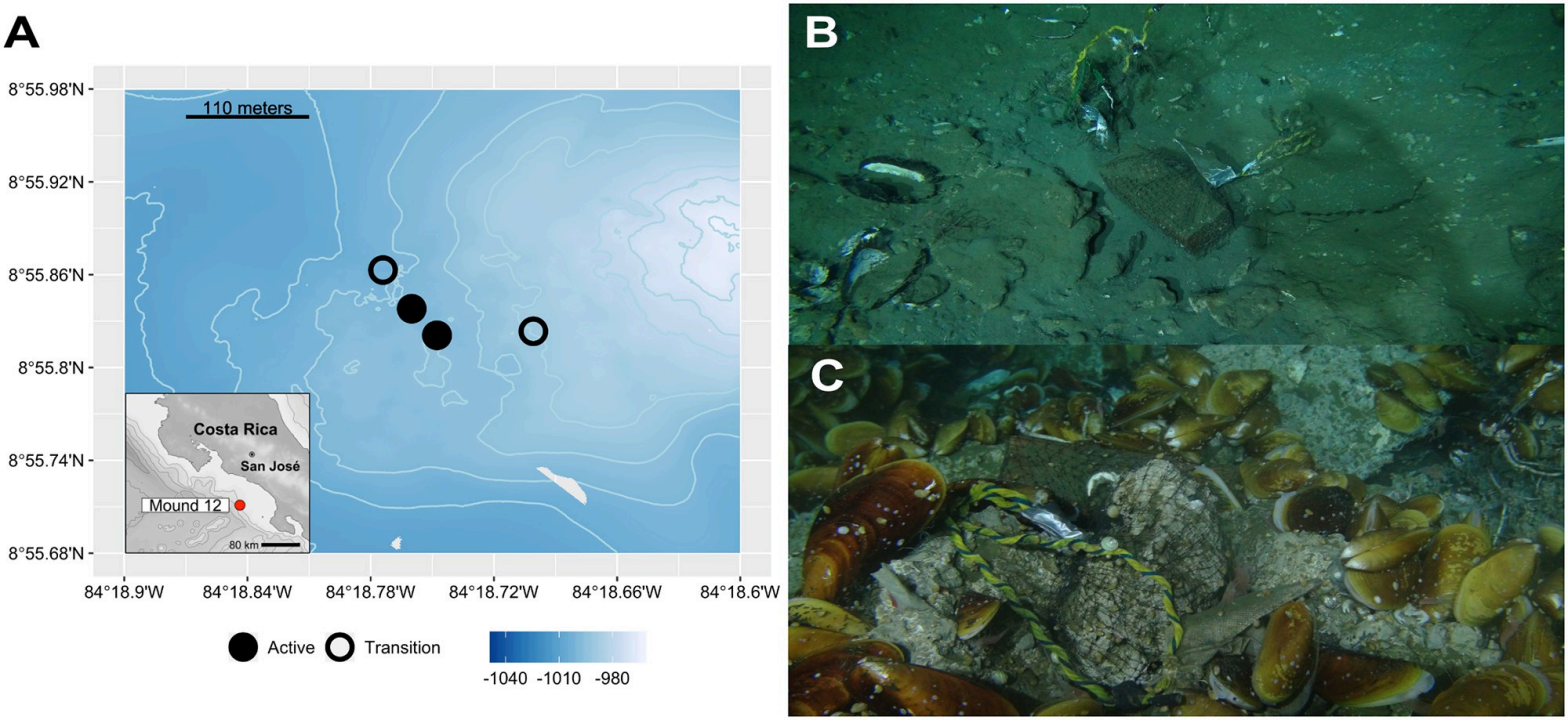
Sampling locations of the experimental substrates and photos *in situ*. Experimental bones, wood, and carbonate rocks were deployed for 7.4 years (2010–2017) at (B) active and (C) transition sites at Mound 12. The map was plotted in R Software using marmap 1.0.6 package ‘getNOAA.bathy()’ function, which queries the ETOPO1 database hosted on the NOAA website in the public domain.

**Fig 2:**
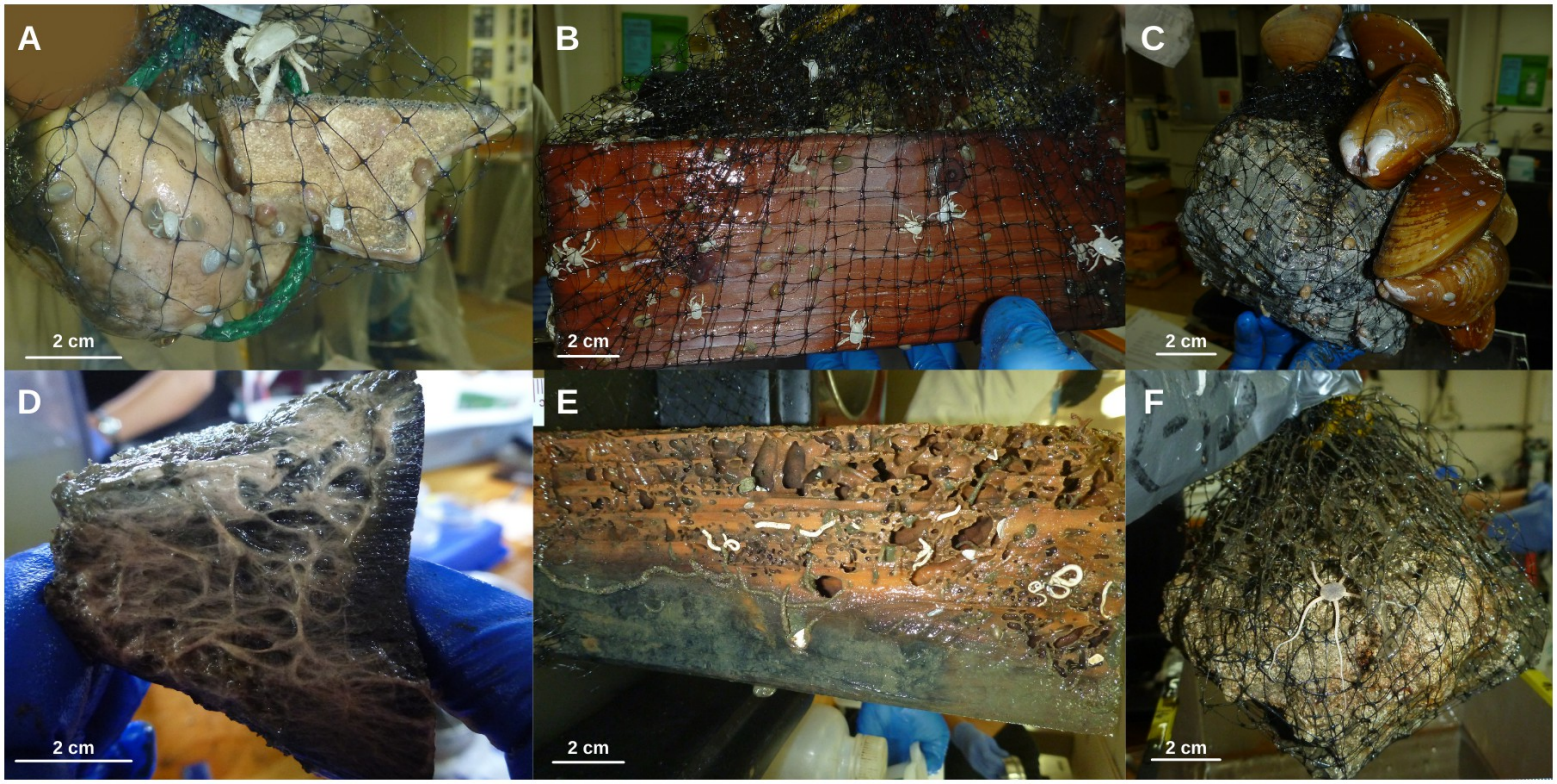
Photos of the experimental substrates upon recovery. Experimental bones, wood, and carbonate rocks were deployed for 7.4 years (2010–2017) at (A-C) active and (D-F) transition sites at Mound 12.

A colonization experiment was performed at Mound 12 on the Pacific margin of Costa Rica to test the interaction between methane seeps and different CBEs (organic falls). Bone (cow bone) and wood blocks (untreated Douglas Fir blocks 10 cm x 10 cm x 25 cm) were deployed in two active (2 bones, 4 wood blocks) and two transition (2 bones, 4 wood blocks) sites in January 2010 on *R/V Atlantis* cruise AT15-59 with the ROV Jason at Mound 12 (Fig 1). Active sites had extensive carbonates colonized by two species of *Bathymodiolus* mussels [41] and other macrofaunal seep species, and transition sites had partly buried carbonates with soft sediment and scattered dead mussels (Fig 1). Within each site, one bone and two wood blocks were deployed within 25–50 cm of each other. Substrates were recovered after 7.4 years with the submersible ALVIN in May 2017 (AT37-13; Fig 2). Substrates were placed into individual containers within Delrin bioboxes on the Alvin basket to avoid cross contamination during recovery. Defaunated carbonate rocks were also deployed for 7.4 years at the same active (n = 4) and transition (n = 4) sites (from rock experiment, see [13]) for comparison of colonization rates among different substrates. These defaunated carbonate rocks had been collected from Mound 12 in 2009. At that time, the fauna were removed upon collection, and the rocks were left out to dry (see [13]). *In situ*, unmanipulated carbonate rocks that were collected in 2017 at active and transition sites were used as control samples for the experiment (see [13]). Here we define control as what we expected the colonizing community to look like at the end of the experiment if full recovery to baseline state was achieved. Here we present the results for the wood and bone experiments, comparing to the results of the rock experiment in Pereira et al. [13].

**Fig 3:**
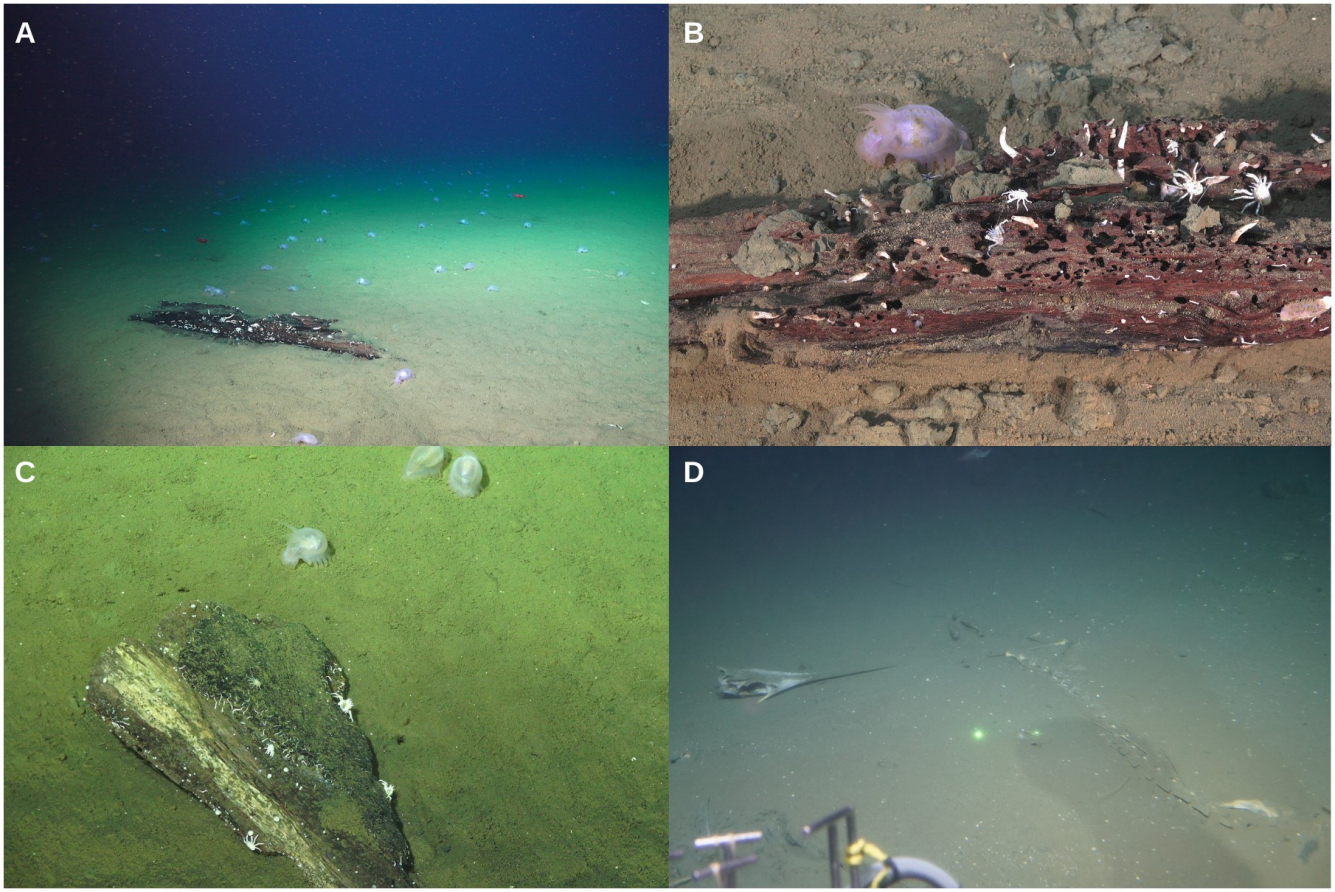
Natural organic falls observed in the vicinities of methane seeps off the Costa Rican margin. (A) Wood found at Jaco Scar (~2,000 m) and a (B) closeup. (C) Wood found at Mound 12 (~1,000 m). (D) Swordfish skeleton found at Quepos Slide (~400 m). Although we only collected samples from the woods, the skeleton observation illustrated here further highlights the common occurrence of natural organic falls in the vicinities of methane seep sites off the Costa Rican margin.

In addition, we have observed natural organic falls in the vicinity of methane seep sites off the Costa Rican margin; these included natural wood pieces at Mound 12 (~1,000 m) and Jaco Scar (~2,000 m), and a swordfish skeleton at Quepos Slide (~400 m) (Fig 3). We collected macrofaunal samples from the natural wood at Mound 12 in 2017 and 2018 and at Jaco Scar in 2017 for taxonomy and isotope analyses following the same methods described below for the experimentally deployed substrates.

### Sample processing

Shipboard and laboratory processing followed Pereira et al. [13]. In summary, substrates were photographed intact upon recovery, the associated fauna was removed from each substrate and its recovery container and sorted and identified to the lowest possible taxonomic level. Tissue subsamples were collected for stable isotope analyses to examine trophic diversity and reliance on chemosynthetic production. Bones and rocks were wrapped in aluminum foil to determine the approximate substrate surface area (in cm^2^) later in the laboratory by dividing the total weight of the foil used to wrap the substrate by the average weight of a 1 cm^2^ piece of foil. Foil wrapping was the most accurate way to estimate surface area due to the rugose, pitted and lumpy nature of the substrates. Initial wood block surfaces were smooth, and area (1047.06 cm^2^) was readily calculated from wood dimensions (10 cm x 10 cm x 25 cm). Substrates were left at room temperature overnight in filtered seawater for additional fauna to crawl out; the fauna were then preserved in 95% ethanol.

For constraining food webs, we collected samples for stable isotopic analyses (δ^13^C and δ^15^N) of the potential food sources: Water samples were collected from Mound 12 at the surface (< 5 m deep) and near-bottom (<15 m above bottom) via Niskin bottles on a CTD Rosette and filtered on glass microfiber filters to characterize particulate organic carbon (POC). Bacteria (mainly *Thioploca*) were scraped off the rocks, and subsamples of each of the rock, wood and bone substrates were powdered using a ceramic mortar and pestle.

At Scripps Institution of Oceanography, the preserved samples were re-sieved and sorted, following Pereira et al. [13]. Species-level IDs based on morphology were considered unreliable since subsequent molecular sequencing showed that some taxa were cryptic species (G.W. Rouse, personal observation), and, thus, Annelida and Mollusca were identified at the family level, Arthropoda (crustaceans) at the order or infraorder level, Cnidaria at the order level, and Echinodermata at the class level. Other macrofauna that occurred in low abundance included Nemertea and Platyhelminthes identified to phylum, and other mollusks (aplacophorans and polyplacophorans) and crustaceans (ostracods and mites). Animals were counted, adding the counts of animals that were removed at sea upon recovery for genetic or isotopic analysis to obtain totals for each substrate. Tissue subsample preparation for isotope analyses followed Pereira et al. [13] and δ^13^C and δ^15^N measurements of tissues, food sources and substrates were carried out at Washington State University (WSU).

### Data synthesis and statistical analyses

Densities of the total macrofaunal community on the different substrates were calculated for a standard surface area of 200 cm^2^ [11, 13], which represents the surface area of an average-sized carbonate rock at Mound 12. As the data were not normally distributed nor showed a homogeneous variance, Kruskal-Wallis tests followed by Dunn’s test using the Benjamini-Hochberg adjustment [42] were performed to check for variability in densities among substrates, and Wilcox tests were performed to check for variability between seepage activity within substrates. Community composition was analyzed by percent composition and density per 200 cm^2^ by taxonomic group. Multi-dimensional scaling of Bray-Curtis dissimilarities, two-way ANOSIM and two-way SIMPER analyses were conducted using Primer v7 [43] after standardizing the data by the total number of individuals and fourth-root transformation.

The stable isotope data were also not normally distributed and did not show homogeneity of variance, thus Kruskal-Wallis tests followed by Dunn’s test using the Benjamini-Hochberg adjustment [42] were performed to check for variability among substrates, and Wilcox tests were performed to check for variability between seepage activity within substrates. Community-level isotope metrics [36] were generated using Stable Isotope Bayesian Ellipses in R (SIBER) package [44], following Pereira et al. [13], to evaluate trophic diversity, trophic redundancy, and niche breadth. Similarities in trophic structure among substrates and seepage activity were determined by computing pairwise ellipse overlaps based on Bayesian posterior estimates using 2 chains of 20,000 iterations with a burn-in of 1,000 and thinning of 10 [45].

## Results

**Fig 4:**
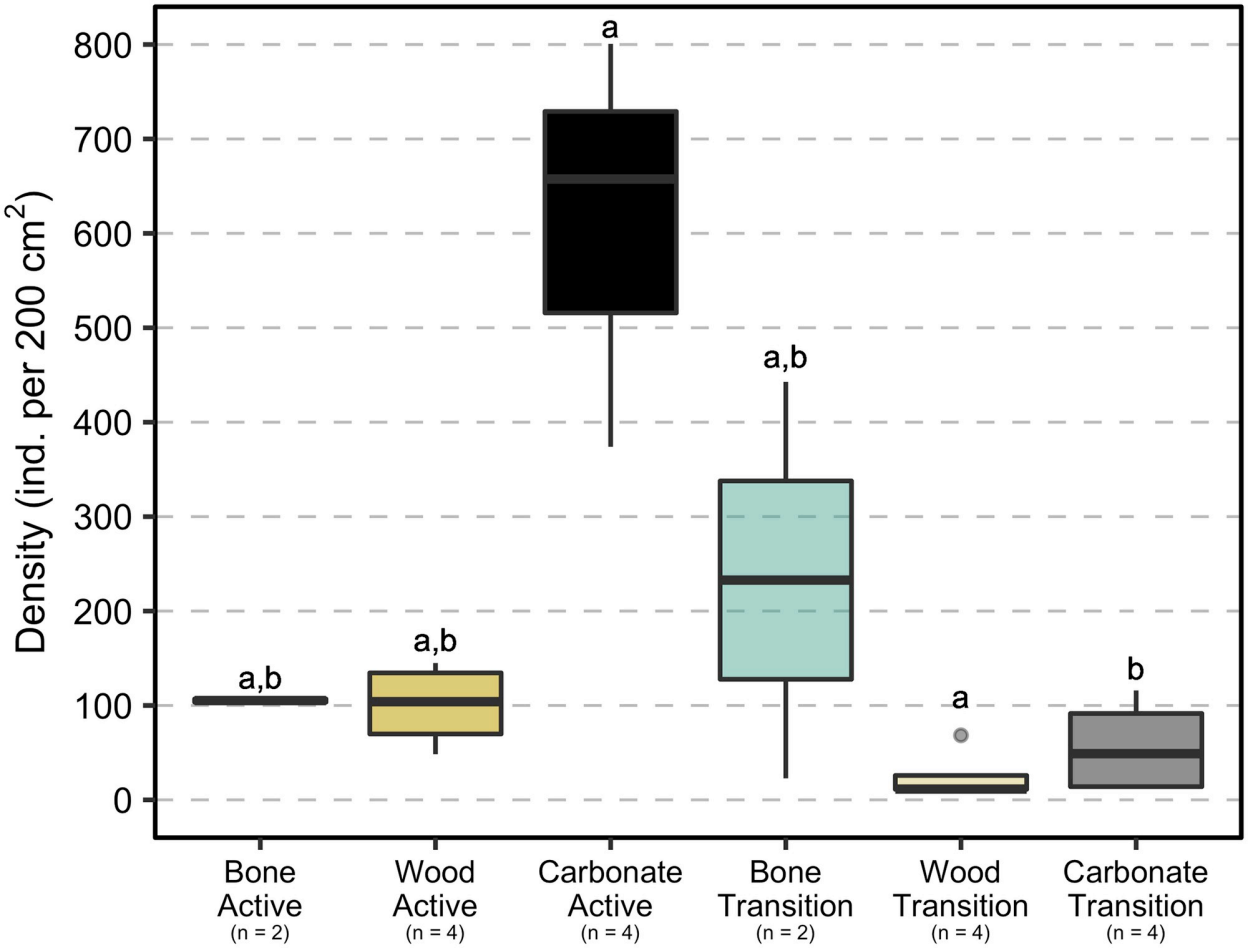
Density of macrofaunal colonizers on the experimental substrates. Densities (individuals per 200 cm^2^) of the macrofaunal invertebrate community on experimental bones, wood, and carbonate rocks deployed for 7.4 years (2010–2017) at active (n = 2 bones, 4 woods, and 4 carbonate rocks) and transition (n = 2 bones, 4 woods, and 4 carbonate rocks) sites at Mound 12. Boxplots visualize five summary statistics: the mean, two hinges (the 25^th^ and 75^th^ percentiles), and two whiskers (extend from the hinge to the largest/smallest value at 1.5x the inter-quartile range), as well as outlying points individually. Data points are available in S1 File.

Average densities at active sites on wood (100 ± 23 ind./200 cm^2^) and bone (105 ± 3 ind./200 cm^2^) were lower than on carbonate rock (610 ± 123 ind./200 cm^2^). At transition sites, the density on bone (233 ± 210 ind./200 cm^2^) was higher than on wood (26 ± 14 ind./200 cm^2^) and rock (57 ± 26 ind./200 cm^2^) (Fig 4). However, the density values were not significantly different among substrates at either active ($\chi_{2}^{2}=5.4$, p = 0.07) or transition sites ($\chi_{2}^{2}=4.0091$, p = 0.13), possibly due to the low sample size and great variability among replicates (S1 Fig, S2 Table). Macrofaunal densities were not significantly different between active and transition sites for the colonizing communities on deployed bone (W = 2, p = 1.00) and wood (W = 15, p = 0.06), in contrast to observations for carbonate rock, for which density was more than 10x higher at active than at transition sites (W = 12, p = 0.05).

**Fig 5:**
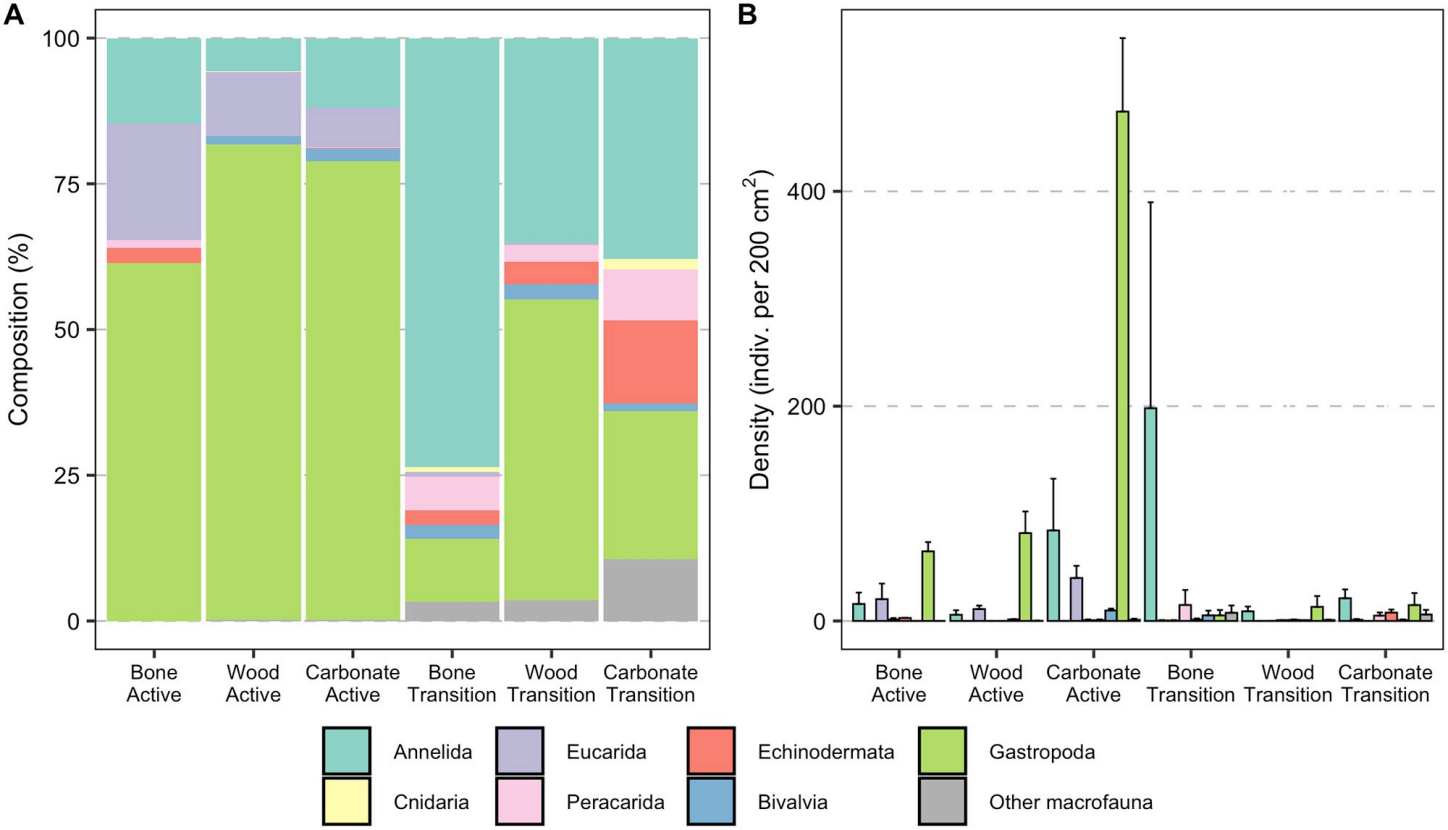
Community composition and density of macrofaunal colonizers on the experimental substrates. (A) Composition (%) and (B) average density (indiv. per 200 cm^2^) of macrofaunal invertebrates on experimental bone, wood, and carbonate rock deployed for 7.4 years (2010–2017) at active and transition sites at Mound 12.

**Fig 6:**
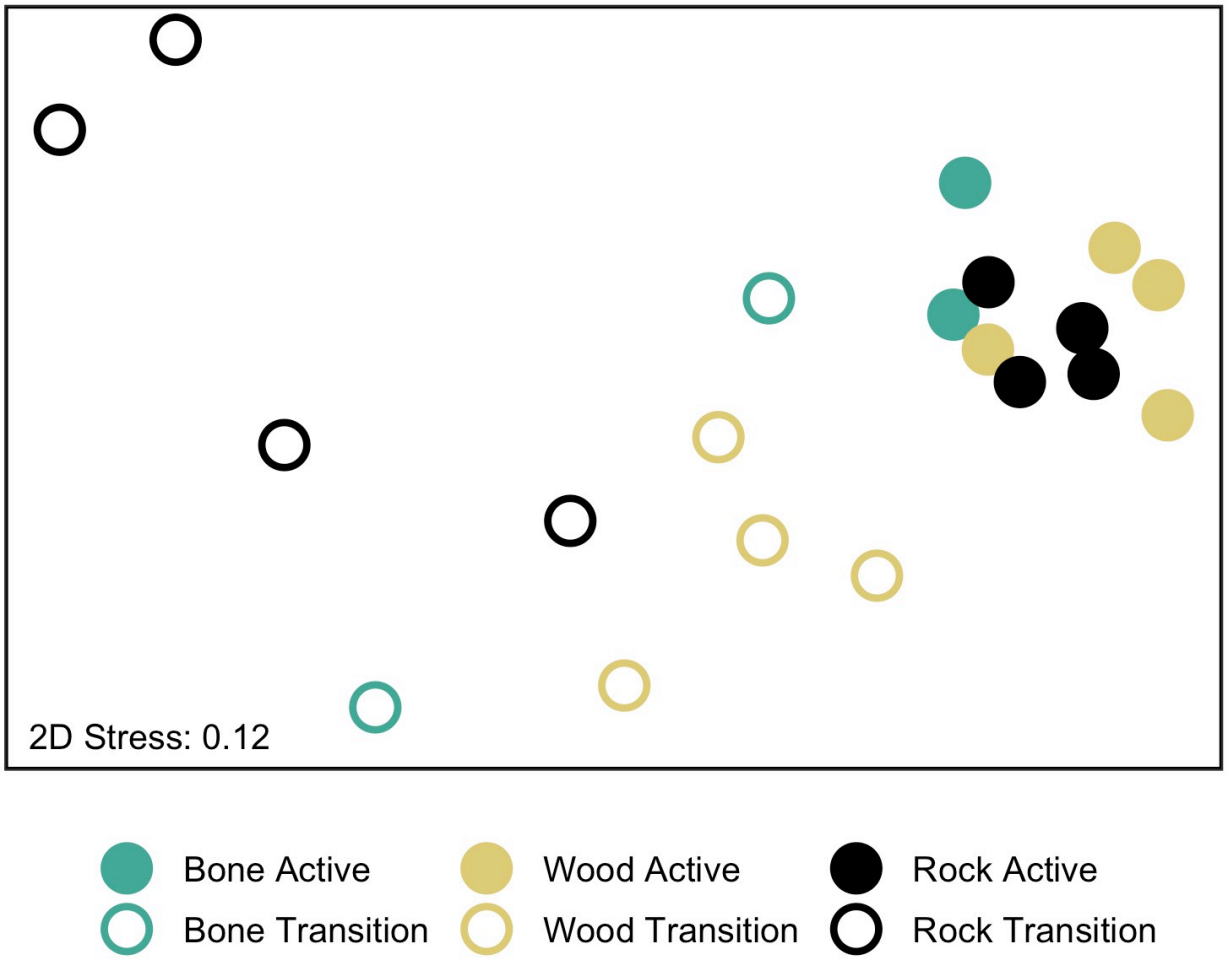
MDS of macrofaunal colonizers on the experimental substrates. Multi-dimensional scaling analysis of macrofaunal invertebrate community composition on experimental cow bone, wood, and carbonate rock deployed for 7.4 years (2010–2017) at active (filled circles) and transition (open circles) sites at Mound 12.

Substrate type did not affect the assemblage composition of the colonizing communities (Two-Way ANOSIM, Global R = 0.389, p = 1.00; Figs 5 and 6), but seepage activity did (Two-Way ANOSIM, Global R = 0.81, p = 0.001). Gastropods (snails and limpets) were the most successful colonizers on all substrates at active sites (78.74% on rocks, 61.33% on bones, and 81.53% on wood; Fig 5A), including mainly Provannidae, Hyalogyrinidae, Lepetodrilidae, Neolepetopsidae and Pyropeltidae (Table 1). Although the density of gastropods on bone and wood were lower than on rocks at active sites (Fig 5B), provannids, lepetodrilids, neolepetopsids and pyropeltids showed high abundance on all three substrates, as did anomurans, mainly yeti crabs (Table 1). Annelids, mainly ampharetids, dorvilleids and hesionids, were the most successful colonizers at transition sites on bone (73.55%) and rock (37.89%), but not on wood, where snails accounted for 42.43% and annelids, mainly hesionids, dorvilleids, and amphinomids were 35.14% of the total macrofauna (Fig 5A). The taxa contributing to dissimilarity between colonizers of active versus transition sites on all substrates combined were more abundant mainly at active sites (namely Provannidae = 6.22%, Anomura = 5.45%, Lepetodrilidae = 5.43%, Neolepetopsidae = 3.52%, Pyropeltidae = 3.43%, Mytilidae = 3.41%, and Ampharetidae = 3.37%), but some taxa were more abundant at transition sites (Ophiuroidea = 5.60%, Serpulidae = 4.54%, and Hesionidae = 3.78%; SIMPER, average dissimilarity = 74.84). We did not find the bone-eating annelid *Osedax* on the bones in our 7-year experiment, and only one *Xylophaga* sp., the wood-borer bivalve, individual was found on one wood block at the transition site despite the fact that the wood blocks at transition sites were heavily burrowed, reflecting past *Xylophaga* activity (Fig 2E).

**Table 1:** Ten most abundant taxa of macrofaunal colonizers on the experimental substrates.

**Active sites**					
**Bone**	**%**	**Wood**	**%**	**Carbonate rock**	**%**
Lepetodrilidae^1^	38.67	Lepetodrilidae^1^	39.22	Lepetodrilidae^1^	33.61
Anomura^1^	20.00	Provannidae^1^	20.04	Provannidae^1^	27.58
Pyropeltidae^1^	8.00	Skeneidae	13.14	Ampharetidae^1^	8.01
Provannidae^1^	6.67	Anomura^1^	10.90	Neolepetopsidae^1^	7.89
Neolepetopsidae^1^	6.67	Neolepetopsidae^1^	4.85	Anomura^1^	6.79
Ampharetidae^1^	5.33	Pyropeltidae^1^	3.38	Hyalogyrinidae	5.99
Dorvilleidae	5.33	Dorvilleidae	3.28	Pyropeltidae^1^	3.54
Cirratulidae	2.67	Mytilidae	1.19	Mytilidae	1.69
Ophiuroidea	2.67	Ampharetidae^1^	0.90	Phyllodocidae	1.31
Hesionidae	1.33	Hyalogyrinidae	0.62	Hesionidae	0.84
	97.33		97.52		97.26
**Transition sites**					
**Bone**	**%**	**Wood**	**%**	**Carbonate rock**	**%**
Hesionidae^2^	22.31	Provannidae	38.48	Ophiuroidea	14.29
Dorvilleidae	9.92	Hesionidae^2^	18.67	Neolepetopsidae	11.80
Ampharetidae	8.26	Dorvilleidae	4.76	Serpulidae	9.94
Lacydoniidae	7.44	Lepetodrilidae	4.19	Amphipoda	6.21
Phyllodocidae	6.61	Neolepetopsidae	4.00	Aplacophora	4.97
Tanaidacea	4.96	Ophiuroidea	3.81	Phyllodocidae	4.35
Serpulidae	4.13	Amphinomidae	2.67	Cataegidae	4.35
Capitellidae	4.13	Amphipoda	2.48	Lepetodrilidae	4.35
Provannidae	4.13	Skeneidae	2.10	Hesionidae^2^	3.73
Amphinomidae	3.31	Cataegidae	2.10	Lacydoniidae	3.11
	75.21		83.24		67.08

Top ten taxa of macrofaunal colonizers on the experimental substrates. given as percent of the total density for cow bones, woods, and carbonate rocks deployed at active and transition sites at Mound 12 for 7.4 years (2010–2017).

^1^ Taxa present on all three substrates at active sites.

^2^ Taxa present on all three substrates at transition sites.

**Fig 7:**
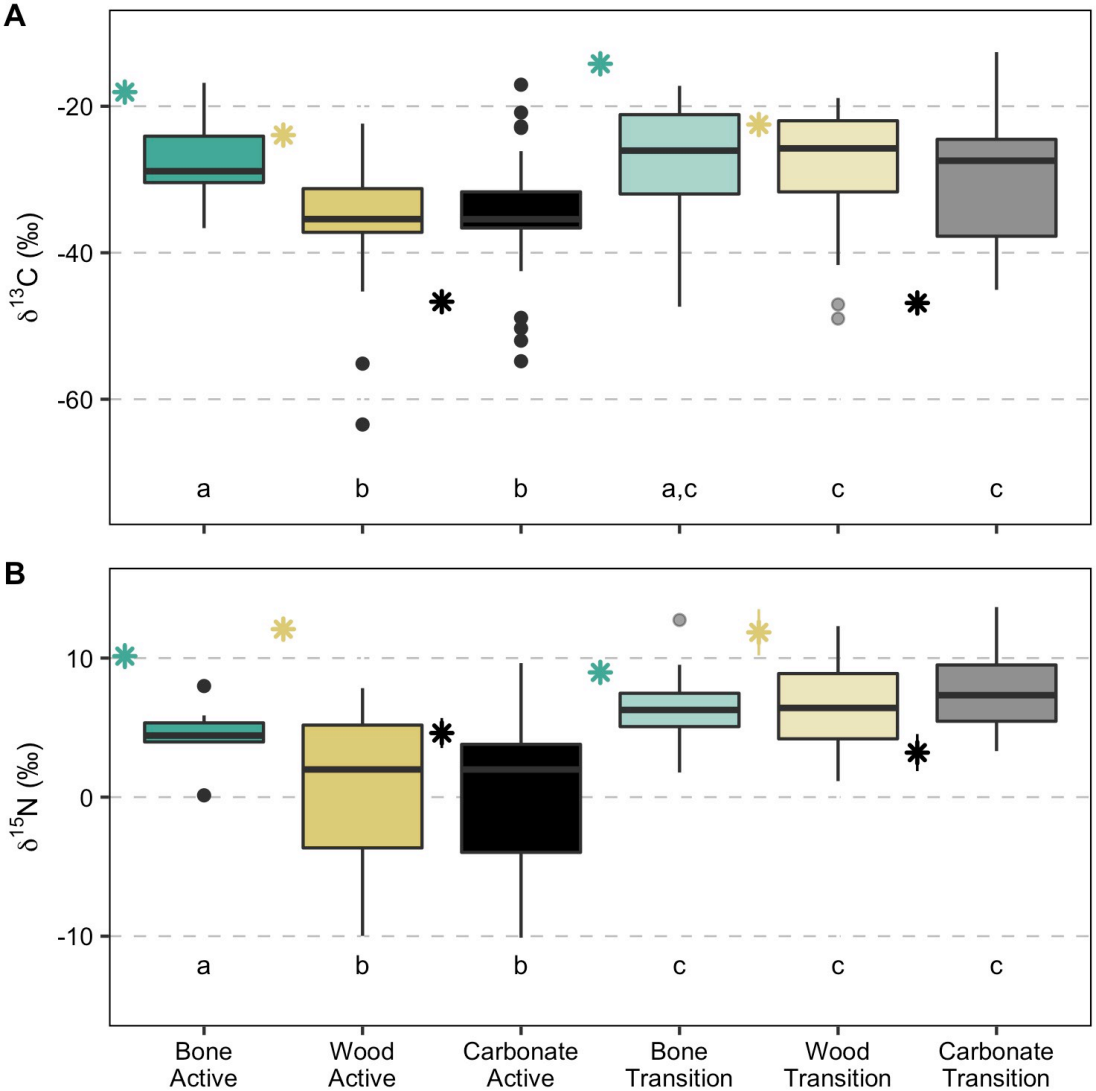
Isotopic composition of macrofaunal colonizers on the experimental substrates. (A) δ^13^C and (B) δ^15^N values (‰) of macrofauna invertebrates colonizing experimental bones, wood and carbonate rocks deployed for 7.4 years (2010–2017) at active and transition sites at Mound 12 and mean (± 1 standard deviation) isotopic composition of each substrate at active and transition sites (stars). Boxplots represent five summary statistics: the mean, two hinges (the 25^th^ and 75^th^ percentiles), and two whiskers (extend from the hinge to the largest/smallest value at 1.5x the inter-quartile range), as well as outlying points individually. Isotope data are available in S1 File.

Mean isotopic C and N composition of the macrofaunal assemblage on experimental hard substrates deployed for 7.4 years (Fig 7) was not significantly different among the three substrates at transition sites (δ^13^C: $\chi_{2}^{2}=0.7463$, p = 0.69, δ^15^N: $\chi_{2}^{2}=1.9216$, p = 0.38). At active sites, the average assemblage C and N isotopic composition varied among different substrates (δ^13^C: $\chi_{2}^{2}=11.3710$, p = 0.003, δ^15^N: $\chi_{2}^{2}=6.2096$, p = 0.04). Assemblages on bones had higher δ^13^C and δ^15^N values than on rock (δ^13^C: z = 6.2193, p = 0.002, δ^15^N: z = 2.4563, p = 0.007) and wood (δ^13^C: z = 3.1359, p = 0.003, δ^15^N: z = 2.1756, p = 0.01), but no difference was observed between wood and rock macrofaunal isotope composition (δ^13^C: z = -0.0165, p = 1.00, δ^15^N: z = -0.3423, p = 0.37).

The macrofaunal community on experimental wood exhibited 8.3‰ and 4.2‰ lower δ^13^C and δ^15^N values, respectively, at active sites than at transition sites on wood (δ^13^C: W = 240, p < 0.0001, δ^15^N: W = 226, p < 0.0001; Fig 7). Animals on bone had 1.8‰ higher δ^15^N values at transition sites (W = 41.5, p = 0.03), but no difference was observed for δ^13^C values (W = 74, p = 0.68).

Isotopic compositions of major taxa were not significantly different among substrates at both active and transition sites, except for annelids that had 2.7‰ lower δ^15^N values on bone than on wood at transition sites (z = -2.6944, p = 0.01).

**Fig 8:**
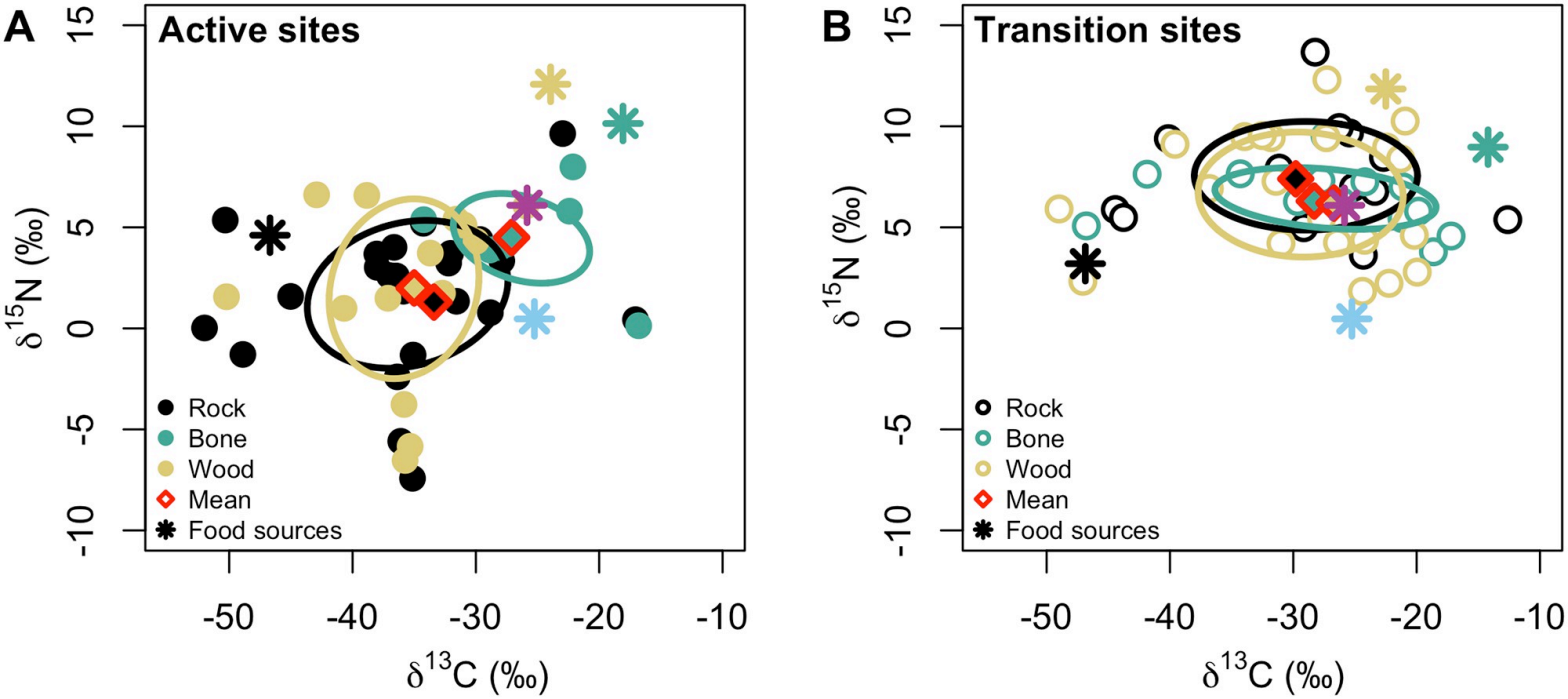
Trophic niche diversity of macrofaunal colonizers on the experimental substrates. Dual isotope plots reflecting macrofaunal invertebrate community trophic niche diversity based on corrected standard ellipse area on *in situ* carbonate rocks (black) and bone (green) and wood (yellow) deployed for 7.4 years (2010–2017) at Mound 12 at (A) active and (B) transition sites. Each point represents the average for one species. Diamonds represent the mean isotopic values for the macrofaunal invertebrate community on each substrate. Stars represent the mean isotopic values for food sources: rock (black), bone (green), wood (yellow), bacteria (blue), and POC (pink). Error bars were omitted for simplicity. Data are available in S1 File.

**Fig 9:**
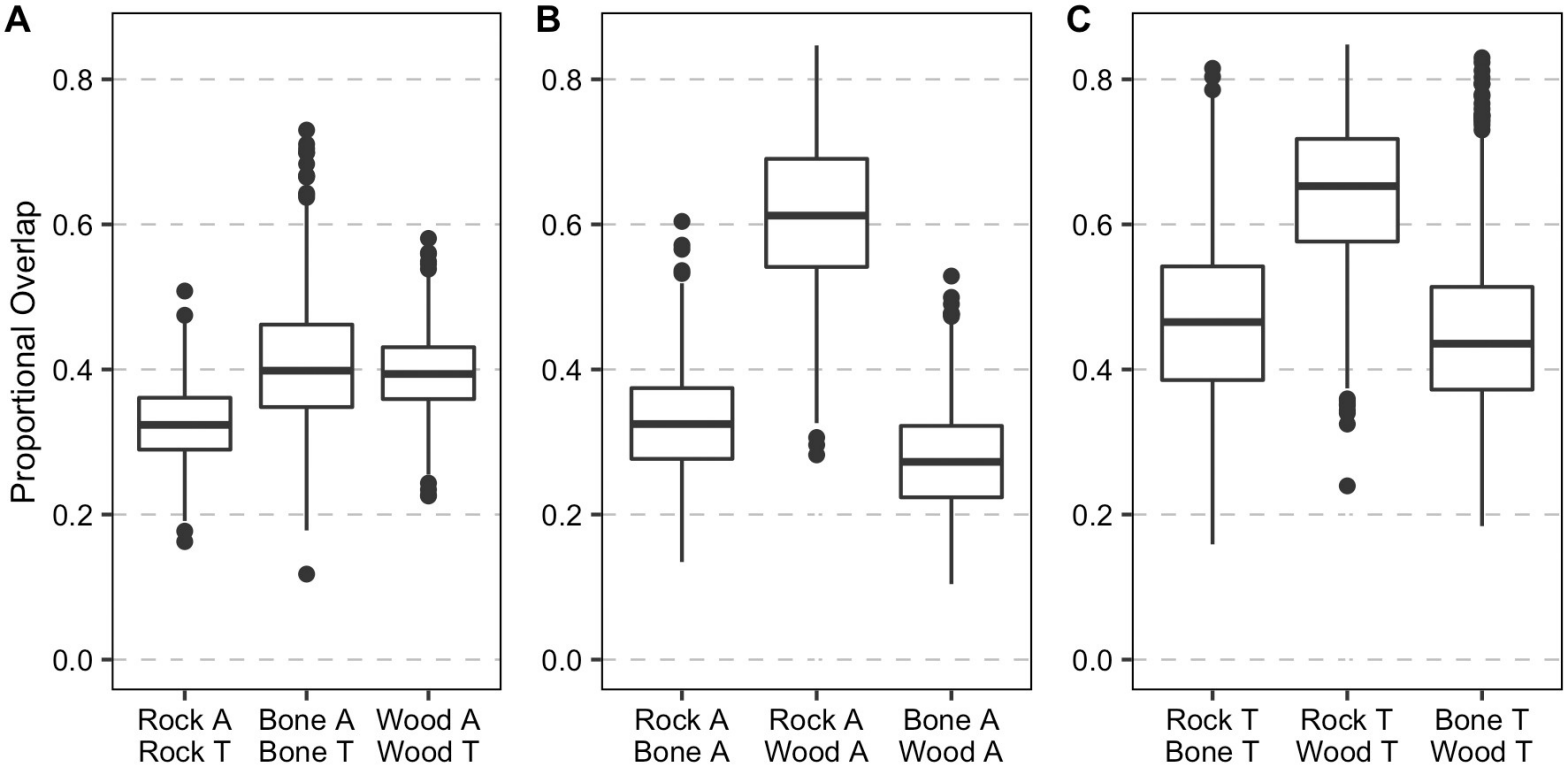
Trophic niche overlap of macrofaunal colonizers on the experimental substrates. Proportional overlap between carbon and nitrogen isotopic niche areas of macrofaunal invertebrates on experimental carbonate rocks (from Pereira et al. [13]), bone and wood deployed at Mound 12 at active and transition sites for 7.4 years (2010–2017). (A) Comparisons between seepage activity within substrate, (B) Comparisons among substrates at active sites, (C) Comparisons among substrates at transition sites. A: Active site, T: Transition site. Boxplots visualize five summary statistics: the median, two hinges (the 25^th^ and 75^th^ percentiles), and two whiskers (extend from the hinge to the largest/smallest value at 1.5x the inter-quartile range), as well as outlying points individually.

The macrofaunal assemblage on wood and rock at active sites exhibited greater trophic diversity than at transition sites, while the opposite trend was observed for the assemblage on the bones (SEAc; Fig 8, S2 Table). The δ^15^N range, possibly reflecting the heavy influence by local N_2_ fixation, was higher at actives sites for all three substrates, as was niche breadth (CD; S2 Table). Trophic redundancy based on mean nearest neighbor distance (MNND) was greater at transition than active sites for rocks and bones, but not for wood (S2 Table). Within substrates under different seepage regimes, niche overlap between seepage activity levels was greater for the colonizers on wood and bone than on carbonate rocks (Fig 9A).

Colonizers on bone and wood had a lower niche diversification at the base of the food web (δ^13^C range) and variable N sources (δ^15^N range) than those on carbonate rocks (S2 Table). This pattern was observed independent of seepage activity. Bone and wood colonizers showed lower trophic diversity than those on carbonate rocks at active sites, but wood assemblages had greater trophic diversity among the three substrates at transition sites. Macrofaunal assemblages on bones had lower trophic diversity than those on wood and rock (Fig 8, S2 Table). Niche overlap between assemblages on different substrates was greater at transition sites than at active sites, except between rock and wood (Fig 9).

Animals colonizing the natural wood collected in 2017 at a transition site at Jaco Scar (~2,000 m) included agglutinated foraminifera, ampharetid and maldanid polychaetes, neolepetopsid limpets, and nemerteans, and the wood-eating bivalve *Xyloredo* sp. The average δ^13^C value of colonizers on the natural wood (-25.6 ± 2.3‰) was not statistically different than on the experimentally deployed wood at Mound 12 (-29.4 ± 1.8‰; W = 74, p = 0.55), but δ^15^N was 2x lower (Natural wood: 2.6 ± 0.6‰, Experimental wood: 6.6 ± 0.7‰; W = 18, p = 0.006).

## Discussion

Our initial hypothesis that seepage activity would override the relative importance of the substrate itself, with macrofaunal communities on rocks (methane seeps), bones (animal falls) and wood (wood falls) being more similar under active seepage, and less similar at transition sites was mostly supported although with some caveats. Indeed, seepage activity had a strong influence on community composition at active sites, where assemblages on bone, wood, and rock were dominated by seep species, with similar trophic niches especially between wood and rock. In contrast, the assemblages on the organic substrates at transition sites were less similar among substrates and resembled natural organic falls (although missing organic-fall specialists) than seep communities, but trophic niche overlap among substrates was high. Below we discuss our hypothesis and its caveats in detail, and the linkages between organic falls and methane seeps off Costa Rica.

### Organic-fall colonizers at seep transition zones

At transition sites, the community composition on wood and bone was similar to natural wood- and whale-fall community composition, which rely on decay of the organic substrates, with high abundance of mainly annelids, and a few peracarid crustaceans and echinoderms (Fig 5A) (see [20, 46]). Unfortunately, some basal food sources may be missing in our isotopic analyses, given that some macrofauna isotopic values do not fall within the range expected for the food source isotopic values we have, and, thus, we could not perform mixing models analyses. However, the higher carbon and nitrogen values at transition sites (Fig 8) reflect less use of methane-derived production and locally fixed nitrogen. Interestingly, macrofauna on wood, carbonate and bone at transition sites seem to be feeding on identical food sources (most likely POC and chemosynthetic bacteria utilizing sulfide from organic-matter decay) (Fig 8) as suggested by the high niche overlap among substrates relative to active sites (Fig 9). This is notable, given that the different substrates shared fewer taxa at transition than at active sites (i.e., lower overlap of taxa) (Table 1).

Bones at transition sites were dark-colored indicating reducing conditions [23] and covered in white bacterial mat (Fig 2D). This reducing state has been seen before on an implanted gray-whale carcass on the California margin after 6.8 years at the seafloor in a sulfophilic stage indicated by the presence of thick white and yellow bacterial mats [18], and on a whale vertebrae from a natural whale fall in the Southwest Atlantic, where the bone was not colonized by *Osedax* and was intact with chemosynthetic bacterial mats [47], considered by the authors to be an intermediate reducing state [20]. The intact state of our colonization bones after 7 years is not a surprise since bone preservation is increased in the absence of *Osedax* [12].

Hesionid and dorvilleid polychaetes were the dominant families on our bones at transition sites after 7.4 years. Previous studies reported that whale-fall macrofaunal communities are dominated by these families of polychaetes, as well as capitellids, including many species of dorvilleids especially from the genus *Ophryotrocha* and *Parougia* [23, 47–49]. Our observation suggests that these annelids are not only successful colonizers of whale falls in the short-term but also in the long-term. Animals on natural bones in an intermediate reducing state relied on carbon derived from free-living chemosynthetic bacteria on the bone [20], supporting our hypotheses that animals were feeding on chemosynthetic bacteria on our bones.

Woods blocks at transition sites were lightly bored by wood-boring bivalves (Fig 2E), although we found only one live *Xylophaga* (see discussion below). The bivalve tunnels provide structural heterogeneity for the colonizing macrofauna [50]. Provannid snails were the dominant taxon on wood at transition sites (38.48%) in our 7.4-year long experiment; they accounted for 20–30% of the gastropods on wood from a previous colonization 10.4-month experiment at Mound 12 [21]. Lepetodrilid limpets showed the opposite pattern; they accounted for ~20% of the wood community after 10.4 months [21] but only 4.2% after 7.4 years. Previous studies reported provannid snails to be less resilient under low seepage activity in the short term [8, 13], and lepetodrilids were more abundant than provannids on the carbonate rocks deployed for 7.4 years (see [13]). Both provannids and lepetodrilids are bacterial grazers or get their nutrition from chemosymbionts [51–55]; provannids appeared to be favored by the bacterial community that established on the wood.

Our woods also showed high abundance of dorvilleid polychaetes and some skeneid snails, as observed in a 15-month colonization experiment [23], sharing the substrate with seep-associated species (e.g., provannid snails and neolepetopsid limpets). Dorvilleids and skeneids most likely feed on bacterial mats on the wood [23], most provannid snails graze on bacteria as mentioned above [55], and neolepetopsid limpets also show flexible diets feeding on bacteria and POC [56, 57]. Our isotope data agrees with these, where animals on wood at transition seem to be feeding on POC and/or bacteria (Fig 8). Nevertheless, the dark coloration on the bottom half of the wood, which was likely buried in sediment, indicate a reducing state. A previous study showed that in the absence of *Xylophaga*, the microbial community can produce hydrogen sulfide and, thus, support a chemosynthesis-based community while degradation is slow [58].

### Active seepage influence on organic-fall colonizers

At active sites, fauna seems to depend less on the organic matter provided by the organic substrates for the following reasons: Although we are missing some food sources, we can infer that there was limited direct use of the bone, wood, or rock substrate organic matter as a food source by the macrofauna at active sites (Fig 8A). If animals were using the substrates as food source, we would expect to see higher macrofaunal isotope values on the organic substrate, especially nitrogen, to reflect the higher values of the substrates compared to chemosynthetic production values (Fig 8). In addition, there was a clear shift in trophic structure from transition to active sites, with lower carbon and nitrogen stable isotope values at active sites, indicating more use of chemosynthetic production (Fig 6).

Lepetodrilids, provannids, anomurans (mainly yeti crabs), and neolepetopsids showed high abundance on wood and bone. These taxa are commonly found associated with hard substrates at active seepage, where seepage activity is a strong determinant of the macrofaunal community composition [11, 13]. Thus, at active sites, it seems that seepage activity overrides the substrate influence, i.e., seep species can colonize wood and bone but not necessarily to feed directly on their organic-rich content. This conclusion is also supported by the similar trophic structure across substrates at active sites, especially for wood and rock (Figs 7–9). In addition, communities on bone and wood attained the same level of dissimilarities between active and transition sites as observed on experimentally deployed carbonate rocks [13], and higher abundance of seep species at active than at transition sites (Table 1), highlighting the stronger influence of seepage activity on community composition across substrates. It seems that the communities associated with organic substrate at active seepage are mainly influenced by the energy available from the seep chemosynthetic production (rather than the organic-rich substrate itself) and the niche dynamics shaped by seepage activity [46, 59, 60].

The similarity between wood and rock could be explained by a delayed wood degradation at active sites. Initial decay of wood in the deep sea usually is mediated by *Xylophaga*, and microbial succession and wood decomposition depend on environmental conditions, especially oxygen [2]. As mentioned above, we did not find *Xylophaga* on the wood at active sites, and seep communities use 2x more oxygen than non-seep communities [61].

Although we did not measure the substrates before the experiment to account for decay, a more rapid decay of the bone could be a possible explanation for the lower niche overlap between macrofauna of the bone and the other two substrates at active sites. However, our bones at active sites did not show signs of degradation, such as black discoloration that indicates reducing conditions [23]. It is possible that this niche difference between bone and wood/rock resulted from the low sample size, as the few species that we collected from the bones for isotope analyses did not reflect use of bone or differ in isotopic composition from the same species on wood and rock (S1 File).

### Lack of organic-fall specialists

Previous experiments with wood deployed for 2 weeks to 2 years suggest that the wood-boring xylophagaid bivalves are early colonizers [23, 46, 62, 63]. The absence of xylophagaids in our 7.4-year experiment, but presence of a single *Xylophaga* individual and burrows in the wood blocks at transition sites reflect past *Xylophaga* activity at earlier successional stages.

This lack of wood specialist species could be explained by a lack of larval supply. Young et al. [23] suggested that local abundance of wood falls is more important to wood specialists than larval transport from distant sources. However, xylophagaid bivalves have been reported from natural wood off Costa Rica, including the ones we collected at Mound 12 and Jaco Scar in 2017 and 2018 and near Cocos Island in 2019 (G.W. Rouse, personal observation, e.g., https://sioapps.ucsd.edu/collections/bi/catalog/M16099/?q=xylophagaidae&image=Any&idx=305, https://sioapps.ucsd.edu/collections/bi/catalog/M16111/?q=xylophagaidae&image=Any&idx=306). Predation also seems unlikely to explain the absence of *Xylophaga* since we did not find common xylophagaid predators, e.g., chrysopetalid, and polynoid polychaetes, gastropods, and flatworms [64, 65] on our wood blocks.

There are then two other possible scenarios that could explain the lack of organic-fall specialists at actively seeping sites: (1) Our wood blocks were at the end of the sulfophilic stage upon recovery [52, 63], and/or (2) seepage activity creates a toxic environment for organic-fall specialists.

*Scenario 1*: In experiments carried out in other regions, wood blocks from shorter experiments were much more degraded than ours. Wood blocks deployed off the coast of Washington for 15 months were crumbling upon recovery due to heavy boring by mostly *Xylophaga oregona* [23], and others deployed in the Southwest Atlantic hosting multiple xylophagaid species lost up to 75% of their volume after almost 2 years [46]. Our wood blocks were not highly degraded, whereas the previously described experiments yielded wood degradation described as ‘crushable by hand’ [23, P.Y.G. Sumida personal communication for Saeedi et al. 46], and ours were not. As lightly-bored wood blocks may represent an early colonization stage where space and organic matter are not limited yet [23], it is then unlikely that our wood blocks at either active or transition sites, were at the end of the sulfophilic stage.

*Scenario 2*: Considering this low degradation state, it is striking that we only found a single *Xylophaga* sp. individual and empty burrows on our wood blocks at transition sites, and none were seen at active sites. As wood-borers are excluded in anoxic conditions [66], seepage activity could create a low-oxygen environment that is toxic for xylophagaid bivalves that were not able to colonize the wood at active sites. At transition sites, where there is less seepage activity, xylophagaid larva were able to settle on the wood blocks but it is unclear why they did not survive. However, in the absence of *Xylophaga*, the wood becomes anoxic due to microbial fermentation [58], possibly impeding new xylophagaid settlement within the 7.4 years of our experiment.

Grupe [21] also hypothesized that nearby seepage could have caused the lack of wood-boring taxa on their wood deployed for 10.5 months at active and transition sites at Mound 12. Surprisingly, other experiments with wood in the Mediterranean did recover high densities of wood-boring bivalves *Xylophaga atlantica* and *Xyloredo ingolfa* on wood pieces deployed for 1 year at a seep [63] and *Xylophaga dorsalis* on ~1–2 years experiments at mud volcanoes [67]. However, on both experiments the deployment sites seem to be heterogenous with soft sediment and some carbonates, or mostly covered in soft sediment with some dead mussels, which we would consider a transition site (see photos in [63, 67]).

In the case of the bones, *Osedax* is considered an early but short-term colonizer [68]. We did not find *Osedax* on our bones nor signs that they were previously there (there were no burrows). Parallel to the wood, a lack of larval supply or bone sources seems unlikely since *Osedax* has also been collected from natural bones and other experimental bones deployed off Costa Rica, including the swordfish skeleton shown in Fig 3D from a low oxygen site (G.W. Rouse, personal observation). It is possible then that seepage is also toxic for *Osedax*. Grupe [21] deployed bones at active and inactive sites in Hydrate Ridge, Oregon, for 10.5 months and found that the bones at inactive sites were colonized by three species of *Osedax*, but not the bones at active sites. However, it is also important to point out that *Osedax* might show bone preference; vertebrae colonized and not colonized by *Osedax* have been collected from the same whale fall in the Southwest Atlantic [47], and *Osedax* were denser on mandibles and thoracic vertebrae and less prevalent on the ribs, sternum, humerus, lumbar vertebrae, and caudal vertebrae on a natural whale fall in Antarctica [69]. Thus, the lack of *Osedax* in our bones could also have been the result of bone characteristics.

Another location where toxic seepage could explain the lack of organic-fall specialists is the Southwest Atlantic, where six landers with bone and wood were deployed for almost 2 years. At the end of the experiment, wood and bones from one of the landers were not colonized by wood-boring bivalves [46] or *Osedax* [70], respectively. Interestingly, as previously mentioned, individuals of the shrimp *Alvinocaris muricola* [24] and the snail *Cordesia* sp. [25], two species previously know only from seeps, were collected from the same wood and bones.

### Organic falls in Costa Rica and linkages to methane seeps

Wood elements can enter the ocean via river systems that carry wood after storms, landslides, hurricanes, and floods. In 2005, hurricane Rita caused major flooding and landslides in Costa Rica, supplying river channels with large quantities of trees [71]. The high wood decay rates in tropical regions (due to higher rate of biological activity, microbial diversity, and year-round warm and moist conditions) allow for breakdown of wood into smaller pieces that can be more easily transported [72]. Tropical storms and associated rapid streamflow also lead to higher wood mobility [72]. The increase in frequency and intensity of extreme climate events will likely increase the transfer rate of wood debris from the land to the marine environments [73]. Moreover, tropical forests in Latin America are threatened by deforestation due to agricultural expansion [74]. In Costa Rica, once the transported wood reaches the ocean, it can be deposited in proximity to the many methane seeps on the continental slope [75].

Similarly, whale falls are commonly found on continental margins along cetacean migration routes [2]. In Costa Rica, humpback whales (*Megaptera novaeangliae*) are the most abundant species of whales, and both humpback and blue whales (*Balaenoptera musculus*) occupy breeding grounds, where they reproduce and give birth during the winter [76, 77]. Dolphins (family Delphinidae) are also an extremely diverse group, with some year-round resident populations [76]. Despite the economic value of these cetaceans to local communities via tourism (whale watching), they remain affected by incidental mortality in fisheries, direct killing for shark bait, and noise pollution [76]. Other carcasses such as large pelagic fishes can also create food-rich islands in the deep sea [78, 79]. In Costa Rica, important fisheries include yellow fin, big-eyed, and skipjack tuna, swordfish, marlin, dolphin and shark [80]. Thus, there is great potential for organic falls in the Costa Rican continental margin, in proximity to methane seeps, supported by our personal observations (Fig 3) Although we used cow bones for this experiment, which have lower lipid contents than whale bones [81], the bone-eating worm, *Osedax* sp., previously thought to be a whale-fall specialist, has been observed colonizing experimentally deployed cow [82], fish [83], alligator [84], bird, and turtle [85] bones, showing that many kinds of bone may support chemosynthetic communities.

The presence of seep fauna such as neolepetopsid limpets on the natural wood, and other seep fauna (e.g., yeti crabs, lepetodrilid limpets, and provannid snails) coexisting with fauna commonly found in organic falls (e.g., dorvilleid and capitellid polychaetes) (see [20]) on the experimentally deployed wood blocks and cow bones supports the hypothesis of organic falls functioning as stepping-stones for dispersal of seep fauna. Transition zones expand the influence of the seep and, as seepage activity might be toxic for organic-fall specialists, it delays the degradation of organic substrates, and seep animals colonizing these at transition zones are not overwhelmed and limited by organic fall specialists [67]. Although these seep species do not seem to be feeding on the organic content of the substrates as do *Osedax* and *Xylophaga*, they are able to colonize them and feed on bacterial mat that establishes on the substrates during the sulfophilic stage. The ability to colonize and reproduce on different substrates increases the likelihood that larva of seep species will find a settlement site [86], thus wood-fall and whale-fall substrates can create ephemeral chemosynthetic habitats that attract a species-rich assemblage with a range of trophic niches, functioning as stepping stones for vent and seep species [18, 20, 87–89]. This habitat heterogeneity can be exploited by both opportunists and endemic fauna (including bone or wood trophic specialists), acting to sustain the persistence and evolution of chemosynthesis-dependent species [90].

## Conclusion

The presence of seep species on wood and bone deployed for 7 years at both active and transition sites, and the similarity in trophic structure among the communities on different substrates, suggest a potential use of hard substrates of other ephemeral chemosynthesis-based ecosystems (wood-falls and whale-falls) as attachment and feeding habitat, functioning as stepping stones for seep fauna even at later successional stages as hard substrate. At active sites, seepage activity and derived chemosynthetic production have strong influence in defining the community composition and trophic structure of macrofauna associated with organic substrates. At transition sites, substrate type has greater influence on communities. Our findings support the idea that chemosynthesis-based ecosystems exist along a reducing continuum [91], and, with increasing human interest in deep-sea resources on continental margins, it is important we understand the connections between such ecosystems.

## Supporting information

S1 Fig Density of macrofaunal colonizers on each experimental substrate. Density (individuals per cm^2^) of the macrofaunal invertebrate community on experimental bones, wood, and carbonate rocks (we did not have surface area measurements for one rock at the active site, thus, n = 3 for density measurements, from Pereira et al. 2021) deployed for 7.4 years (2010–2017) at active and transition sites at Mound 12.
(PDF)

S1 Table Terminology and definitions. (PDF)

S2 Table Community metrics for macrofaunal colonizers on the experimental substrates. Community metrics for macrofauna community on bone, wood and carbonate rock deployed for 7.4 years (2010–2017) at active and transition sites at Mound 12. Carbonate rocks data from Pereira et al. (2021). TA: Total area; SEA: Standard ellipse area; SEAc: Corrected standard ellipse area; CD: Mean distance to centroid; MNND: Mean nearest neighbor distance; SDNND: Standard deviation of nearest neighbor distance.
(PDF)

S1 File Data points behind means and standard errors in figures. All raw data is available at the BCO-DMO database (https://www.bco-dmo.org/project/648472). Carbonate rocks data from Pereira et al. (2021) https://doi.org/10.1002/ecs2.3744.
(PDF)
