# Supplementary material for: Does substrate matter in the deep sea? A comparison of bone, wood, and carbonate rock colonizers
Source: PLoS One. 2022 Jul 20;17(7):e0271635. doi: 10.1371/journal.pone.0271635 (PMC9299329; doi:10.1371/journal.pone.0271635)

Pereira et al. Does the substrate matter in the deep sea? A comparison of bone, wood, and carbonate rock colonizers

S1 Figure. Density of macrofaunal colonizers on each experimental substrate. Density (individuals per cm^2) of the macrofaunal invertebrate community on experimental bones, wood, and carbonate rocks (we did not have surface area measurements for one rock at the active site, thus, n = 3 for density measurements, from Pereira et al. 2021) deployed for 7.4 years (2010-2017) at active and transition sites at Mound 12.

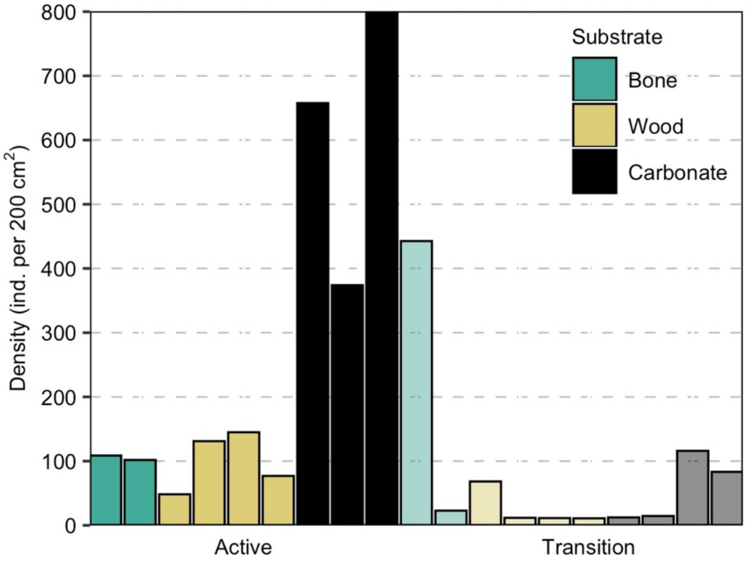

Supplement: S1 Fig — Density (individuals per cm2) of the macrofaunal invertebrate community on experimental bones, wood, and carbonate rocks (we did not have surface area measurements for one rock at the active site, thus, n = 3 for density measurements, from Pereira et al. 2021) deployed for 7.4 years (2010–2017) at active and transition sites at Mound 12. (PDF) [file pone.0271635.s001.pdf]
