# Supplementary material for: Does substrate matter in the deep sea? A comparison of bone, wood, and carbonate rock colonizers
Source: PLoS One. 2022 Jul 20;17(7):e0271635. doi: 10.1371/journal.pone.0271635 (PMC9299329; doi:10.1371/journal.pone.0271635)
Supplement: S1 Table — (PDF) [file pone.0271635.s002.pdf]

Pereira et al. Does substrate matter in the deep sea? A comparison of bone, wood, and carbonate rock colonizers

S1 Table. Terminology and definitions.

Macrofauna	Animals retained on a 300 μm sieve but smaller than 2 cm. This may include juveniles of animals that could be considered megafauna (e.g., yeti crab *Kiwa puravida*).
Seepage activity spatial zonation (referred to as areas or zones)	
Active	'Center' of the seep, with high seepage activity, extensive carbonate rocks, and seep-specialist megafauna such as the yeti crab *Kiwa puravida* and *Bathymodiolus* mussels.
Transition	Area surrounding the active area, where there is less seepage activity. As described in the Methods section: Declines in fluid flux over time and space can create this transition zone surrounding the active area, where bacterial biomass and seep endemic species diminish but the rocks formed during high seepage activity levels persist providing hard substrate for background species. Microbial activity may still persist within the carbonates, and some seep species can still be found on theses rock. Thus, transition zones can be highly diverse, hosting seep and background communities.
Background	Surrounding deep sea with no seepage activity, carbonate rocks, seep megafauna, and bacterial mats.
Site	
Active site	Site within the active zone.
Transition site	Site within the transition zone identified by the presence of scattered seep-specialist megafauna (e.g., yeti crab *Kiwa puravida* and *Bathymodiolus* mussels), shell remains of seep-associated bivalves (e.g., *Bathymodiolus* mussels and *Calyptogena* clams), and partially buried but visible carbonate rocks hosting seep and background species (see definitions below).
Substrates	
Experimental substrates	Bone, wood and/or carbonate rock* experimentally deployed for 7.4 years at active and transition sites at Mound 12.
In situ carbonate rocks*	Unmanipulated carbonate rocks collected in 2017 at active and transition sites at Mound 12. Used as control samples for the experiment, i.e., what we expcted the colonizing community to look like at the end of the experiment.
Species/taxa	
Seep species/taxa	Species/taxa that are more endemic to seeps.

Background species/taxa	Species/taxa that are more commonly associated with background conditions (no seepage).
Communities	
Seep communities	Communities composed mainly by seep species.
Background communities	Communities composed mainly by background species.

* Carbonate rock data from rock colonization experiment in Pereira et al. (2021).
