# Supplementary material for: Does substrate matter in the deep sea? A comparison of bone, wood, and carbonate rock colonizers
Source: PLoS One. 2022 Jul 20;17(7):e0271635. doi: 10.1371/journal.pone.0271635 (PMC9299329; doi:10.1371/journal.pone.0271635)
Supplement: S2 Table — Community metrics for macrofauna community on bone, wood and carbonate rock deployed for 7.4 years (2010–2017) at active and transition sites at Mound 12. Carbonate rocks data from Pereira et al. (2021). TA: Total area; SEA: Standard ellipse area; SEAc: Corrected standard ellipse area; CD: Mean distance to centroid; MNND: Mean nearest neighbor distance; SDNND: Standard deviation of nearest neighbor distance. (PDF) [file pone.0271635.s003.pdf]

Pereira et al. Does substrate matter in the deep sea? A comparison of bone, wood, and carbonate rock colonizers

S2 Table. Community metrics for macrofaunal colonizers on the experimental substrates.

	Sample size	Food sources	Trophic levels	Trophic diversity			Trophic redundancy and niche breadth		
		$\delta^{13}C$ range	$\delta^{15}N$ range	TA	SEA	SEAc	CD	MNND	SDNND
**Active sites**									
Bone	2	17.43	7.86	54.51	36.85	42.99	4.72	2.49	2.48
Wood	4	24.28	13.15	176.58	84.14	91.15	6.49	2.82	2.22
Carbonate rock	4	34.94	17.06	365.03	91.99	96.18	6.97	2.73	2.54
**Transition sites**									
Bone	2	29.57	5.73	99.08	41.25	44.99	7.04	2.62	1.57
Wood	4	29.04	10.47	215.18	80.73	84.98	7.38	2.25	0.99
Carbonate rock	4	31.82	10.05	173.82	76.42	83.36	7.61	3.01	2.71

Community metrics for macrofauna community on bone, wood and carbonate rock deployed for 7.4 years (2010-2017) at active and transition sites at Mound 12. Carbonate rocks data from Pereira et al. (2021). TA: Total area; SEA: Standard ellipse area; SEAc: Corrected standard ellipse area; CD: Mean distance to centroid; MNND: Mean nearest neighbor distance; SDNND: Standard deviation of nearest neighbor distance.
