# Supplementary material for: Does substrate matter in the deep sea? A comparison of bone, wood, and carbonate rock colonizers
Source: PLoS One. 2022 Jul 20;17(7):e0271635. doi: 10.1371/journal.pone.0271635 (PMC9299329; doi:10.1371/journal.pone.0271635)
Supplement: S1 File — All raw data is available at the BCO-DMO database (https://www.bco-dmo.org/project/648472). Carbonate rocks data from Pereira et al. (2021) https://doi.org/10.1002/ecs2.3744. (PDF) [file pone.0271635.s004.pdf]

Pereira et al. Does substrate matter in the deep sea? A comparison of bone, wood, and carbonate rock colonizers

S1 File. Data behind means and standard errors in figures. All raw data is available at the BCO-DMO database (https://www.bco-dmo.org/project/648472). Carbonate rocks data from Pereira et al. (2021) https://doi.org/10.1002/ecs2.3744.

Fig 4 data points: Surface area, number of individuals, and density of macrofaunal colonizers on each experimental substrate.

Substrate	Habitat	Substrate number	Surface area (cm^2)	Count	Density (number of indiv. per 200 cm^2)
Bone	Active	Bone 5	68.13	37	108.62
Bone	Active	Bone 6	74.69	38	101.75
Bone	Transition	Bone 3	41.56	92	442.73
Bone	Transition	Bone 7	254.38	29	22.80
Wood	Active	Wood 9	1047.06	253	48.33
Wood	Active	Wood 10	1047.06	686	131.03
Wood	Active	Wood 13	1047.06	759	144.98
Wood	Active	Wood 14	1047.06	403	76.98
Wood	Transition	Wood 11	1047.06	358	68.38
Wood	Transition	Wood 12	1047.06	61	11.65
Wood	Transition	Wood 15	1047.06	59	11.27
Wood	Transition	Wood 16	1047.06	57	10.89
Rock	Active	Rock 13	125.31	412	657.57
Rock	Active	Rock 14	194.69	364	373.93
Rock	Active	Rock 18	242.81	972	800.63
Rock	Transition	Rock 15	192.19	14	14.57
Rock	Transition	Rock 16	161.56	10	12.38
Rock	Transition	Rock 19	139.69	81	115.97
Rock	Transition	Rock 20	134.38	56	83.35

Fig 5A data points: Taxa percent composition of macrofaunal colonizers on experimental substrates.

**Taxa**	**Percent composition (%)**		
	**Bone Active**	**Wood Active**	**Carbonate Rock Active**
Annelida	14.67	5.71	11.94
Cnidaria	-	0.05	-
Eucarida	20.00	11.04	6.83
Peracarida	1.33	-	0.08
Echinodermata	2.67	0.05	0.08
Bivalvia	-	1.43	2.19
Gastropoda	61.33	81.53	78.74
Other macrofauna	-	0.19	0.13
	**Bone Transition**	**Wood Transition**	**Carbonate Rock Transition**
Annelida	73.55	35.14	37.89
Cnidaria	0.83	-	1.86
Eucarida	0.83	0.37	-
Peracarida	5.79	2.80	8.70
Echinodermata	2.48	3.93	14.29
Bivalvia	2.48	2.62	1.24
Gastropoda	10.74	51.59	25.46
Other macrofauna	3.31	3.55	10.56

Fig 5B data points: Mean ± standard error densities of macrofaunal colonizers on experimental substrates by taxa.

**Taxa**	**Density (number of indiv. per 200 cm^2)**		
	**Bone Active**	**Wood Active**	**Carbonate rock Active**
Annelida	15.89 ± 10.53	5.73 ± 4.27	84.27 ± 48.13
Cnidaria	-	0.05 ± 0.048	-
Echinodermata	2.81 ± 0.13	0.05 ± 0.05	0.68 ± 0.68
Peracarida	1.34 ± 1.34	-	0.68 ± 0.68
Eucarida	20.34 ± 14.47	11.08 ± 3.27	40.00 ± 11.31
Bivalvia	-	1.43 ± 0.37	9.79 ± 1.69
Gastropoda	64.81 ± 8.58	81.80 ± 20.14	474.20 ± 68.42
Other macrofauna	-	0.19 ± 0.19	1.06 ± 1.06
	**Bone Transition**	**Wood Transition**	**Carbonate rock Transition**
Annelida	198.04 ± 191.75	8.98 ± 4.45	21.16 ± 8.22
Cnidaria	0.39 ± 0.39	-	1.10 ± 0.71
Echinodermata	1.18 ± 1.18	1.00 ± 0.24	7.80 ± 2.87
Peracarida	14.83 ± 14.04	0.72 ± 0.21	4.97 ± 2.94
Eucarida	0.39 ± 0.39	0.09 ± 0.09	-
Bivalvia	5.20 ± 4.42	0.67 ± 0.12	0.71 ± 0.71
Gastropoda	5.11 ± 5.11	13.18 ± 10.01	14.79 ± 11.18
Other macrofauna	7.61 ± 6.82	0.91 ± 0.21	6.03 ± 4.30

Fig 7 data points: Mean ± standard error δ^{13}C and δ^{15}N values (‰) of (A) macrofaunal colonizers on experimental substrates, and (B) of the substrate itself.

**Substrate**	**Habitat**	**δ^{13}C (‰)**	**δ^{15}N (‰)**
**A. Isotopic composition of the macrofaunal on the substrates**			
Bone	Active	-27.5 ± 1.6	4.5 ± 0.6
Wood	Active	-35.5 ± 1.3	0.8 ± 0.8
Carbonate rock	Active	-34.8 ± 1.2	0.5 ± 0.7
Bone	Transition	-28.5 ± 2.5	6.4 ± 0.6
Wood	Transition	-27.8 ± 1.3	6.3 ± 0.5
Carbonate rock	Transition	-29.5 ± 2.2	7.4 ± 0.6
**B. Isotopic composition of the substrates**			
Bone	Active	-18.1 ± 0.9	10.1 ± 0.9
Wood	Active	-23.94 ± 0.3	12.1 ± 0.6
Carbonate rock	Active	-46.7 ± 0.7	4.6 ± 1.1
Bone	Transition	-14.2	9.0
Wood	Transition	-22.5 ± 0.8	11.8 ± 1.7
Carbonate rock	Transition	-46.9 ± 1.69	3.2 ± 1.3

Fig 8 macrofaunal data points (circles): Mean ± standard error $\delta^{13}C$ and $\delta^{15}N$ values (‰) of macrofaunal colonizers on experimental substrates by species (or lowest taxonomic level possible).

Substrate	Habitat	Species	$\delta^{13}C$ (‰)	$\delta^{15}N$ (‰)
Bone	Active	Amphipoda	-22.1	7.9
Bone	Active	Cirratulidae	-16.8	0.1
Bone	Active	*Kiwa puravida*	-27.5 ± 1.7	4.2 ± 0.2
Bone	Active	*Lepetodrilus* sp.	-28.9	3.9
Bone	Active	Ophiuroidea	-28.0	4.0
Bone	Active	*Ophryotrocha* sp.	-22.4	5.8
Bone	Active	*Provanna laevis*	-30.4 ± 1.1	4.3± 0.1
Bone	Active	*Pyropelta* sp.	-34.2 ± 2.4	5.4 ± 0.5
Wood	Active	*Archinome levinae*	-37.1	1.5
Wood	Active	*Bathymodiolus earlougheri*	-35.7 ± 0.2	-6.5 ± 0.8
Wood	Active	*Bathymodiolus nancyschneideri*	-35.8 ± 0.6	-3.8 ± 0.8
Wood	Active	*Kanoia myronfeinbergi*	-38.8	6.6
Wood	Active	*Kiwa puravida*	-30.9 ± 2.6	5.1 ± 1.1
Wood	Active	*Lepetodrilus* sp.	-30.0 ± 0.8	4.3 ± 0.7
Wood	Active	*Lepetodrilus shannonae*	-25.94	6.1
Wood	Active	*Neoamphitus* sp.	-32.7	1.7
Wood	Active	*Neolepetopsis* sp.	-50.2 ± 4.9	1.6 ± 0.6
Wood	Active	Polynoidae	-35.3	-5.8
Wood	Active	*Provanna laevis*	-33.7 ± 1.7	3.7 ± 0.6
Wood	Active	*Pyropelta* sp.	-40.7 ± 2.6	1.0 ± 1.1
Wood	Active	Shrimp	-42.9 ± 20.5	6.6 ± 1.0
Wood	Active	Terebellidae	-31.6	5.4
Carbonate rock	Active	*Amphisamytha* sp.	-45.0 ± 9.8	1.6 ± 0.5
Carbonate rock	Active	*Archinome levinae*	-37.0 ± 3.1	2.6 ± 1.8
Carbonate rock	Active	*Bathymodiolus earlougheri*	-35.1 ± 1.1	-7.4 ± 0.7
Carbonate rock	Active	*Bathymodiolus nancyschneideri*	-36.1 ± 0.2	-5.6 ± 0.6
Carbonate rock	Active	*Brada* sp.	-35.9	1.8
Carbonate rock	Active	Chiton	-38.0	3.0
Carbonate rock	Active	Cirratulidae	-32.0	3.7
Carbonate rock	Active	Cocculinidae	-52.0	0.1
Carbonate rock	Active	*Gyptis robertscrippsii*	-31.6	1.3
Carbonate rock	Active	Hesionidae	-27.9 ± 7.0	3.3 ± 0.4
Carbonate rock	Active	*Idas* sp.	-28.8 ± 6.1	0.775 ± 4.755
Carbonate rock	Active	*Kiwa puravida*	-29.7 ± 1.56	4.4 ± 2.1

Carbonate rock	Active	*Lacydonia* sp.	-32.2	3.2
Carbonate rock	Active	*Laminatubus joycebrooksi*	-48.9	-1.3
Carbonate rock	Active	*Lepetodrilus* sp.	-36.4	2.6
Carbonate rock	Active	*Neoamphitrite* sp.	-34.3	5.2
Carbonate rock	Active	*Neoamphitus* sp.	-38.0	3.7
Carbonate rock	Active	*Neolepetopsis* sp.	-50.3	5.3
Carbonate rock	Active	Polychaeta	-17.1	0.4
Carbonate rock	Active	Polynoidae	-35.1	-1.3
Carbonate rock	Active	*Provanna laevis*	-36.6 ± 3.0	4.0 ± 1.2
Carbonate rock	Active	Pyropelta	-36.4	-2.4
Carbonate rock	Active	Shrimp	-23.0	9.6
Bone	Transition	Aplacophora	-24.2 ± 0.5	7.2 ± 5.5
Bone	Transition	*Archinome levinae*	-41.8	7.6
Bone	Transition	Hydroid	-29.7	6.3
Bone	Transition	Neoamphitus	-21.2	5.4
Bone	Transition	Nereididae	-18.6	3.8
Bone	Transition	*Nicomache* sp.	-21.1	7.0
Bone	Transition	Ophiuroidea	-17.2	4.6
Bone	Transition	*Pliocardia krylovata*	-34.3	7.6
Bone	Transition	*Provanna laevis*	-27.7	7.3
Bone	Transition	Sabellidae	-27.4	9.5
Bone	Transition	Serpulidae	-46.9 ± 0.6	5.1 ± 0.1
Bone	Transition	Terebellidae	-19.8	5.8
Bone	Transition	Trichobranchidae	-26.1	6.3
Wood	Transition	Amphipoda	-24.2	4.4
Wood	Transition	Aplacophora	-22.4	9.0
Wood	Transition	*Archinome levinae*	-27.3 ± 3.4	9.4 ± 0.7
Wood	Transition	Chiton	-36.8 ± 4.9	6.9 ± 0.7
Wood	Transition	*Gyptis robertscrippsi*	-31.8	9.4
Wood	Transition	Hesionidae	-21.3 ± 0.7	8.4 ± 0.2
Wood	Transition	*Hyalogyrina* sp.	-22.2	2.2
Wood	Transition	*Kanoia myronfeinbergi*	-39.6	9.1
Wood	Transition	*Kiwa puravida*	-24.4	1.8
Wood	Transition	*Lacydonia* sp.	-31.0	4.2
Wood	Transition	*Lepetodrilus* sp.	-27.7 ± 1.8	5.4 ± 0.8
Wood	Transition	Maldanidae	-33.9	9.5
Wood	Transition	Nemertea	-20.9	10.3
Wood	Transition	*Neoamphitrite* sp.	-31.4	7.3
Wood	Transition	*Neolepetopsis* sp.	-49.0	5.9

Wood	Transition	Ophiuroidea	-26.4 ± 1.8	4.2 ± 1.2
Wood	Transition	Platyhelminthes	-19.9 ± 0.6	2.8 ± 1.5
Wood	Transition	Polynoidae	-27.3	12.3
Wood	Transition	*Provanna laevis*	-47.1	2.3
Wood	Transition	Terebellidae	-32.5	9.5
Wood	Transition	*Xyloredo* sp.	-20.2 ± 0.5	4.6 ± 0.7
Carbonate rock	Transition	Amphipoda	-25.4	9.7
Carbonate rock	Transition	*Archinome levinae*	-22.7	8.5
Carbonate rock	Transition	Cirratulidae	-44.4	5.9
Carbonate rock	Transition	*Eulepetopsis* sp.	-24.3	3.6
Carbonate rock	Transition	Flabelligeridae	-31.1	7.9
Carbonate rock	Transition	Gammaridae	-26.3	9.9
Carbonate rock	Transition	Isopoda	-29.1	5.0
Carbonate rock	Transition	*Kanoia myronfeinbergi*	-40.1 ± 0.2	9.4 ± 0.2
Carbonate rock	Transition	Nemertea	-25.1	7.0
Carbonate rock	Transition	Ophiuroidea	-23.4 ± 3.1	6.8 ± 1.4
Carbonate rock	Transition	Phyllodocidae	-28.2	13.7
Carbonate rock	Transition	*Pyropelta* sp.	-12.6	5.4
Carbonate rock	Transition	Serpulidae	-43.8 ± 1.3	5.4 ± 0.2

Fig 8 food sources data points: $\delta^{13}C$ and $\delta^{15}N$ values (‰) of bacteria and particulate organic carbon (POC) at Mound 12.

**Food source**	**$\delta^{13}C$ (‰)**	**$\delta^{15}N$ (‰)**
Bacteria (*Thioploca*)	-25.2	0.5
POC	-25.8	6.1

See values for bone, wood, carbonate and mean community with Fig 7 data points above.
